# Visualization analysis of mitochondrial dynamics in heart failure based on bibliometrics: Trends, hotspots, and topics

**DOI:** 10.1097/MD.0000000000037598

**Published:** 2024-03-15

**Authors:** Sihan Jia, Yanjie Lian, Sinai Li, Hongxu Liu, Juju Shang

**Affiliations:** a Department of Cardiology, Beijing Hospital of Traditional Chinese Medicine, Capital Medical University, Beijing, China; b Department of Cardiology, Capital Medical University, Beijing, China; c Department of Cardiology, Beijing Institute of Traditional Chinese Medicine, Beijing, China.

**Keywords:** bibliometrics, CiteSpace, heart failure, mitochondrial dynamics, visual analysis

## Abstract

This study aimed to conduct a visual analysis of the relevant literature on mitochondrial dynamics in heart failure, explore the research progress, frontier topics, and development trends in this field, and provide references for the study concerning mitochondrial dynamics in the prevention and treatment of heart failure. The Web of Science was searched from inception to October 1, 2023 to identify relevant English literature on mitochondrial dynamics in heart failure. Bibliometric methods were utilized to statistically analyze the eligible literature, and CiteSpace 6.2.R5 software was employed to visualize data such as countries of publication, institutions, authors, and keywords. A total of 1755 Science Citation Index articles were included. The global publication volume showed an increasing trend year by year, with China and the United States having the most publications, and the United States displaying the highest centrality in publications. As revealed by keyword and citation analyses, the research hotspots and frontiers in this field mainly included the pathogenesis of heart failure, mitochondrial dynamics markers, mitochondrial quality control, and potential therapeutic targets for heart failure. Research on mitochondrial dynamics in heart failure is under vigorous development. It is a development trend in this research field to explore the differential gene expression and molecular mechanisms of targeted treatment in the mitochondrial dynamics in heart failure, which will contribute to the formulation of new strategies for the prevention and treatment of heart failure.

## 1. Introduction

Heart failure (HF) is a syndrome characterized by structural and/or functional abnormalities of the heart, which can result in impaired ventricular contraction and/or relaxation and lead to circulatory dysfunction.^[1]^ Generally speaking, HF is associated with high incidence, hospitalization, and mortality rates,^[2]^ and has become a global public health issue seriously threatening human health.^[3]^ Although evidence-based treatment has reduced the incidence of HF to some extent, the readmission rate remains unfortunately high.^[4]^ As reported in some research, myocardial energy metabolic disorder is a major mechanism underlying HF,^[5]^ and mitochondrial dynamics play a crucial role in maintaining energy metabolism.^[6]^

Mitochondria are the important organelles involved in energy metabolism in human cells. The heart is one of the organs with the highest energy demands in the human body. In mature cardiac cells, mitochondria account for 40% of the cell volume. And the adenosine triphosphate (ATP) supply generated by mitochondrial energy metabolism is responsible for the normal contraction of cardiac cells and the proper conduction of cardiac electrical signals. Therefore, mitochondrial dysfunction can directly affect the function of cardiac cells.^[7]^ Notably, mitochondrial abnormalities include impaired electron transport chain activity, increased reactive oxygen species (ROS) production, altered substrate utilization, abnormal mitochondrial dynamics, and changes in the ion homeostasis.^[8]^ Among them, mitochondrial dynamics balance is the foundation for maintaining normal mitochondrial structure and function.^[9]^ Under physiological conditions, mitochondria undergo continuous fission and fusion to maintain the normal mitochondrial quantity and structural functionality. This ensures the stability of mitochondrial morphology, structure, abundance, and network organization, and this characteristic of mitochondria is referred to as mitochondrial dynamics.^[10]^

Mitochondria are composed of an inner membrane and an outer membrane. Substrates can be oxidized through the respiratory chain in the inner membrane, thereby releasing electrons. During this process, a higher concentration of hydrogen ions is generated between the inner and outer membranes, creating a membrane potential across the mitochondrial membrane. A normal membrane potential is a prerequisite for mitochondrial oxidative phosphorylation and ATP production.^[11]^ However, excessive mitochondrial fission can lead to fragmented mitochondrial morphology, which thus reduces the number of hydrogen ions stored between the inner and outer membranes, decreases the membrane potential, and reduces energy production. On the other hand, the highly fused mitochondria form a network-like structure, and their coupling enhances the degree of substrate oxidation, increases the number of hydrogen ions between the inner and outer membranes, raises the membrane potential, and increases energy production.^[12]^ In this regard, the delicate balance between mitochondrial fusion and fission is crucial for cell survival and optimal function.

CiteSpace, developed by Professor Chaomei Chen from Drexel University in the United States, is a bibliometric software widely recognized and utilized in recent years.^[13–15]^ Therefore, in this study, CiteSpace 6.2.R5 software was used to visualize a knowledge map of research on mitochondrial dynamics in the field of HF based on relevant literature retrieved from the Web of Science database. This approach contributes to more intuitively demonstrating the current research status, development trends, research hotspots, and potential directions, so as to provide references and insights for future studies in this field.

## 2. Materials and methods

### 2.1. Data source and search strategy

The data source for bibliometric analysis in this study was the Web of Science Core Collection. Web of Science Core Collection has been extensively applied in bibliometric research because it provides a comprehensive overview of relevant information, including publications, citations, authors, references, and keywords.^[16]^ In this study, the WoS database was adopted for the primary search using the search terms of TS = (“heart failure” OR “HF”) AND TS = (“Mitochondrial dynamics” OR “Mitochondrial fission” OR “mitochondrial fusion”) from database inception to October 1, 2023. The document types were limited to “Article” and “Review,” whereas the publication language was limited to “English.” The search was conducted on October 1, 2023. Irrelevant literature was excluded from the search results. Thereafter, the full records of the search results, including titles, authors, sources, abstracts, affiliations, document types, keywords, cited references, and publication years, were exported in a text file “download_**.txt” for subsequent analysis. Additionally, to ensure the accuracy and reliability of the research data, 2 researchers independently screened relevant literature and collected data. Any disagreement between them was solved by a third party involved in the decision-making process.

### 2.2. Analysis methods

CiteSpace 6.2.R5 software was employed for visual analysis of authors, institutions, countries, journals, and keywords. Relevant visual maps were generated to analyze the current status, frontier hotspots, and development trends of research on mitochondrial dynamics in HF.

## 3. Results

### 3.1. Distribution of literature

**Figure 1.**
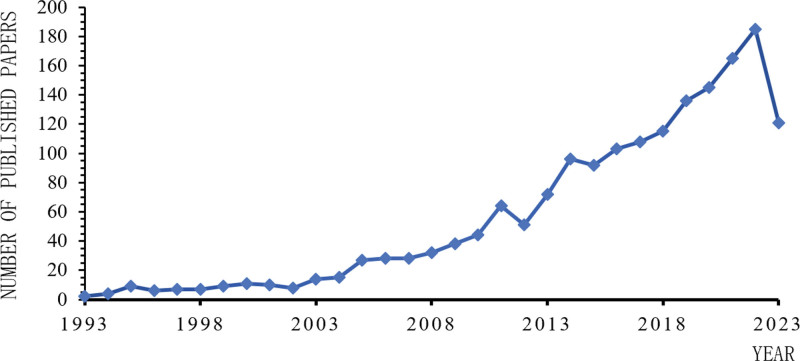
Trend of annual publication volume.

A total of 1755 articles were included in the analysis. The annual publication trend is shown in Figure 1. As observed, research on mitochondrial dynamics in HF was first published in 1993. Based on the annual growth rate of publications, the entire timeline was roughly divided into 3 stages: an initial stage I (1993–2003), a steady development stage II (2004–2011), and a rapid development stage III (2012–2023). According to Figure 1, the number of publications in Stage I was relatively low, with an average of about 10 articles being published per year. In Stage II, the number of publications became stable and increased slightly. In Stage III, the number of publications increased year by year, with over 50 articles being published every year, reaching a peak of 185 articles in 2022. This indicates that research on mitochondrial dynamics in HF is under rapid development in recent years and has gained increasing attention from experts and scholars, exhibiting a promising research outlook.

### 3.2. Collaboration network analysis

#### 3.2.1. Distribution of authors.

**Figure 2.**
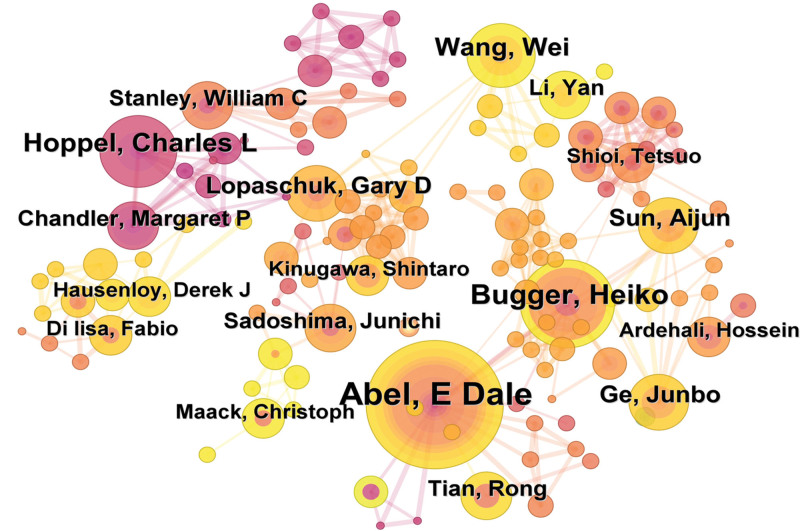
Author collaboration chart.

Network map visualization can provide information about the influential research teams and potential collaborators, which can help researchers establish the collaborative relationships.^[17]^ By using the CiteSpace software, an author collaboration map was generated (Fig. 2). Table 1 shows the authors with a publication frequency of ≥ 7 in the retrieved literature regarding mitochondrial dynamics in HF. The authors, ranked in a descending order, were Abel, E. Dale; Bugger, Heiko; Hoppel, Charles L; Chattipakorn, Nipon; Wang, Wei; Chattipakorn, Siriporn C; Sun, Aijun; Lopaschuk, Gary D; and Ge, Junbo, indicating their significant contributions to investigating the role of mitochondrial dynamics in HF.

**Table 1:** Authors with a publication frequency of ≥7.

Rank	Author	Year	Publications
1	Abel, E Dale	2008	16
2	Bugger, Heiko	2010	11
3	Hoppel, Charles L	2007	9
4	Chattipakorn, Nipon	2020	9
5	Wang, Wei	2018	8
6	Chattipakorn, Siriporn C	2020	8
7	Sun, Aijun	2016	7
8	Lopaschuk, Gary D	2013	7
9	Ge, Junbo	2016	7

#### 3.2.2. Distribution of countries/institutions.

**Figure 3.**
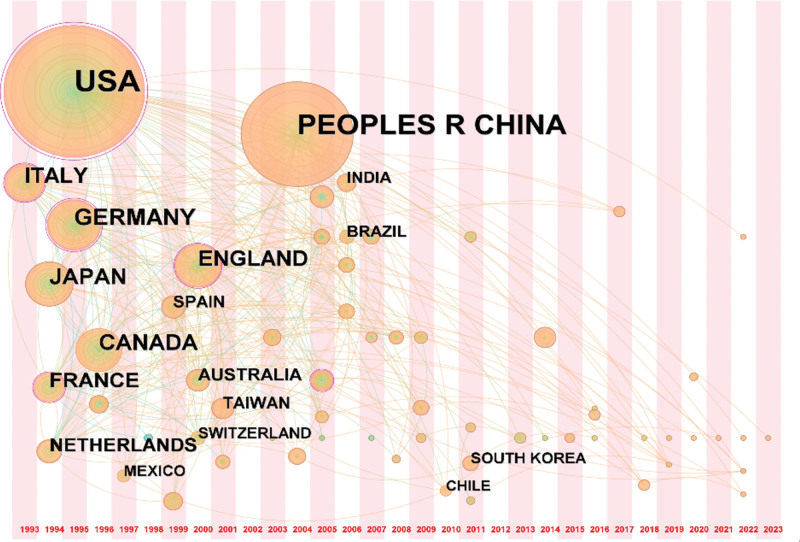
Research country co-occurrence map.

By adopting the CiteSpace software, co-occurrence maps of countries and institutions were plotted. As observed from the maps, there were multiple countries and institutions involved in the research on mitochondrial dynamics in HF. According to the results of this study, 63 countries participated in the research related to mitochondrial dynamics in HF. The collaboration relationships among different countries are presented in Figure 3, while the top 10 countries/regions in terms of the publication volume are listed in Table 2.

**Table 2:** Number of national publications.

Country	Publications	Percentage	Year
USA	690	0.78	1992
PEOPLES R CHINA	451	0.03	2004
GERMANY	138	0.21	1995
JAPAN	117	0.02	1994
CANADA	109	0.09	1996
ENGLAND	100	0.17	2000
ITALY	91	0.11	1993
FRANCE	81	0.12	1994
NETHERLANDS	48	0.02	1994
AUSTRALIA	34	0.06	2000

**Figure 4.**
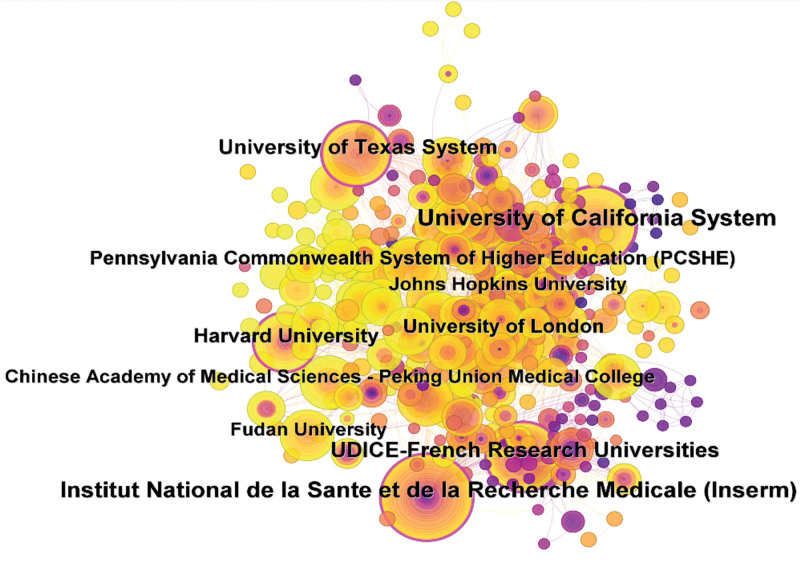
Mapping of institutional cooperation.

Typically, the top 10 countries were mainly distributed in North America, Asia, and Europe. The country with the highest number of publications was the United States (n = 690, 39.32%), followed by China (n = 451, 25.70%) and Germany (n = 138, 7.86%). These 3 countries accounted for more than half of the total publication volume. Later, a timeline graph (Fig. 3) was constructed based on the publication volume and the relationships among the 63 countries according to the publication time. It was seen from Figure 3 that, the United States had the earliest initiation, the highest contribution, and the largest publication volume in this field, with a centrality of 0.78, which ranked the first place. Additionally, there were numerous constructive collaborations between countries. For example, the United States collaborated closely with China, the United Kingdom, Germany, Italy, and Japan, while China collaborated more with the United States, Canada, and the United Kingdom. These countries had high levels of research expertise and developed economies. The collaboration map of institutions is displayed in Figure 4, and the top 5 institutions with regard to the publication volume are listed in Table 3. These institutions were the University of California from the United States (n = 70, 3.99%); the National Institute of Health and Medical Research from France (n = 61, 3.48%); the University of Montpellier from France (n = 49, 2.79%); the University of Texas from the United States (n = 41, 2.34%); and the Harvard University from the United States (n = 36, 2.05%). Three of these 5 institutions were from the United States, and 2 were from France. Moreover, the top 5 institutions also ranked high in centrality, with the University of California from the United States having the highest intermediary centrality of 0.27, indicating that research institutions in the United States may play a dominant or bridging role in the international collaborations in this research field.

**Table 3:** Top 5 research institutions in terms of the number of publications and centrality.

Rank	Institution	Year	Quantity	Centrality
1	University of California System	1995	70	0.27
2	Institut National de la Sante et de la Recherche Medicale (Inserm)	1999	61	0.19
3	UDICE-French Research Universities	1997	49	0.15
4	University of Texas System	2010	41	0.1
5	Harvard University	1999	36	0.11

#### 3.2.3. Journal publication volume.

By adopting the VOSviewer software, journals with a publication volume exceeding 20 articles were analyzed. There were altogether 16 journals identified, as shown in Table 4. Among them, the journal “Journal of Molecular and Cellular Cardiology” had the highest publication volume (79 articles), while the journal “Circulation” had the highest impact factor (IF = 39.918). This indicates that research papers in this field have received wide attention and recognition from the internationally authoritative journals, highlighting the quality and academic influence of the research in this area.

**Table 4:** Number of magazine publications.

Rank	Journals	The number of published papers
1	JOURNAL OF MOLECULAR AND CELLULAR CARDIOLOGY	79
2	AMERICAN JOURNAL OF PHYSIOLOGY-HEART AND CIRCULATORY PHYSIOLOGY	63
3	CARDIOVASCULAR RESEARCH	56
4	CIRCULATION RESEARCH	51
5	INTERNATIONAL JOURNAL OF MOLECULAR SCIENCES	45
6	CIRCULATION	44
7	PLOS ONE	39
8	FRONTIERS IN CARDIOVASCULAR MEDICINE	32
9	FRONTIERS IN PHARMACOLOGY	27
10	OXIDATIVE MEDICINE AND CELLULAR LONGEVITY	26
11	JOURNAL OF CELLULAR AND MOLECULAR MEDICINE	26
12	JOURNAL OF THE AMERICAN HEART ASSOCIATION	25
13	JOURNAL OF THE AMERICAN COLLEGE OF CARDIOLOGY	25
14	HEART FAILURE REVIEWS	23
15	SCIENTIFIC REPORTS	22
16	ANTIOXIDANTS & REDOX SIGNALING	20

### 3.3. Keyword analysis

#### 3.3.1. Co-occurrence of keywords.

**Figure 5.**
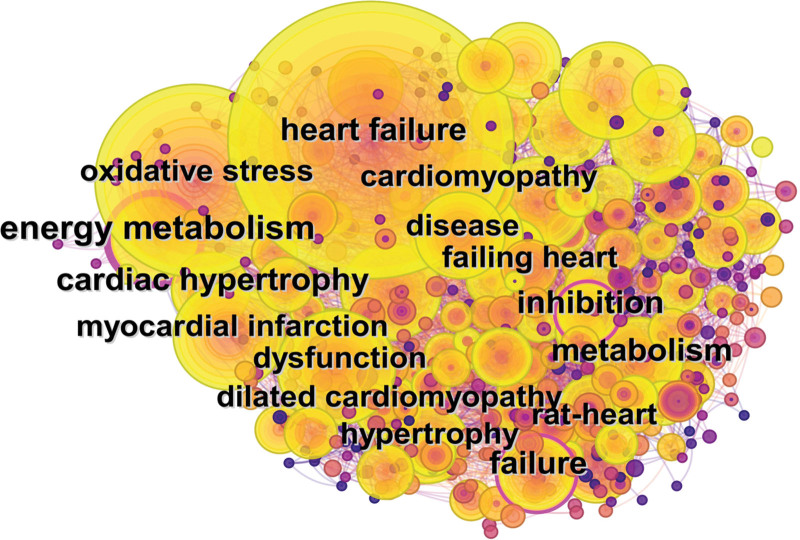
Keyword co-occurrence mapping.

The co-occurrence of keywords can reflect the core research topics and hotspots in the field of mitochondrial dynamics in HF. In Figure 5, a co-occurrence keyword map was generated, and the following analysis was obtained combined with Table 5. The top 20 frequently occurring keywords in this field included heart failure, oxidative stress, functional impairment, myocardial infarction, energy metabolism, expression, triggering, cell apoptosis, metabolism, disease, hypertrophy, mechanisms, ischemia–reperfusion injury, dilated cardiomyopathy, mitochondrial dysfunction, failure, cardiac hypertrophy, fatty acid oxidation, cardiomyopathy, and inhibition. Besides, through comprehensive analysis of these keywords, the research hotspots in the field of mitochondrial dynamics in HF were summarized as follows: regulatory mechanisms of mitochondrial dynamics in HF caused by different etiologies, application of mitochondrial dynamics in mitochondrial dysfunction and energy metabolism in HF, and study on the role of oxidative stress in mitochondrial dynamics in HF.

**Table 5:** High-frequency keywords.

Rank	Keyword	Frequency	Centrality	Rank	Keyword	Frequency	Centrality
1	Heart failure	940	0.13	11	Hypertrophy	126	0.05
2	Oxidative stress	435	0.04	12	Mechanisms	122	0.02
3	Dysfunction	226	0.03	13	Ischemia reperfusion injury	110	0.02
4	Myocardial infarction	195	0.05	14	Dilated cardiomyopathy	104	0.1
5	Energy metabolism	172	0.15	15	Mitochondrial dysfunction	103	0.02
6	Expression	156	0.02	16	Failure	103	0.07
7	Activation	149	0.04	17	Cardiac hypertrophy	102	0.04
8	Apoptosis	142	0.04	18	Fatty acid oxidation	102	0.04
9	Metabolism	129	0.05	19	Cardiomyopathy	92	0.08
10	Disease	128	0.05	20	Inhibition	90	0.05

#### 3.3.2. Keyword cluster analysis.

**Figure 6.**
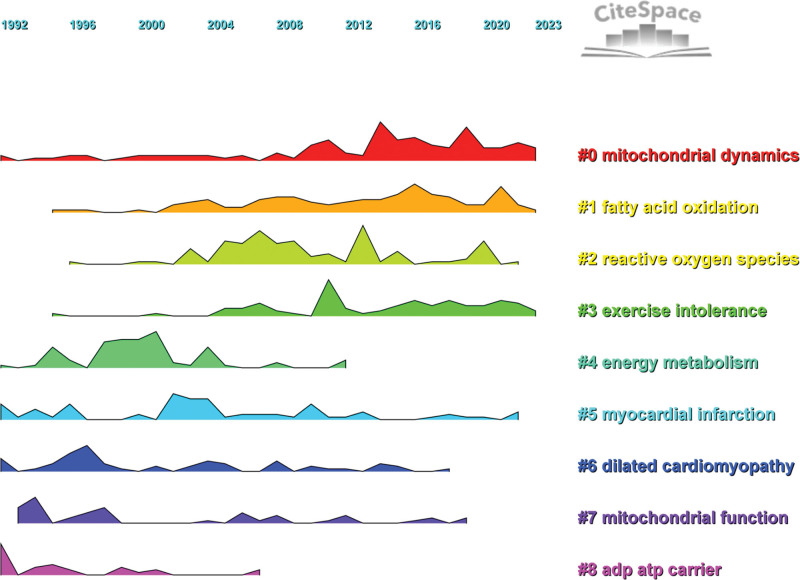
Keyword clustering.

Keyword cluster analysis was carried out with CiteSpace, and a keyword cluster map with a modularity Q of 0.3076 and an average silhouette value S of 0.6733 was obtained, as shown in Figure 6. The cluster structure is significant and reasonable at the thresholds of Q > 0.3 and S > 0.5.^[15]^ According to the keyword cluster map, research on mitochondrial dynamics in HF could be divided into 9 major clusters, namely, mitochondrial dynamics, fatty acid oxidation, ROS, exercise intolerance, energy metabolism, myocardial infarction, dilated cardiomyopathy, mitochondrial function, and adenosine diphosphate ATP carrier. The research directions of these 9 clusters in the field of mitochondrial dynamics in HF were summarized based on the keywords in each cluster, as shown in Table 6.

**Table 6:** Clustering labels.

Number	Frequency	Centrality	Keyword
0	123	0.636	Mitochondrial dynamics; mitophagy; mitochondrial quality control; mitochondrial fission; drp1
1	109	0.542	Fatty acid oxidation; insulin-resistance; lysine acetylation; adipose-tissue; gene-expression
2	100	0.654	Reactive oxygen species; mitochondrial dysfunction; oxidative stress; ischemia reperfusion injury; reperfusion injury
3	77	0.646	Exercise intolerance; sglt2 inhibitors; skeletal muscle; diabetes mellitus; chronic heart failure
4	76	0.775	Energy metabolism; heart failure; creatine kinase; activation; endothelin
5	73	0.68	Myocardial infarction; cardiac function; mice; mitochondrial dysfunction; failure
6	55	0.754	Dilated cardiomyopathy; mouse; cardiac dysfunction; mutant mice; creatine-kinase
7	46	0.778	Mitochondrial function; fatty-acids; cardiac hypertrophy; reactive oxygen species; mitochondrial cardiomyopathy
8	29	0.844	ADP ATP carrier; congestive heart failure; myocarditis; adenine nucleotide translocator; no-donator

#### 3.3.3. Keyword burst analysis.

**Figure 7.**
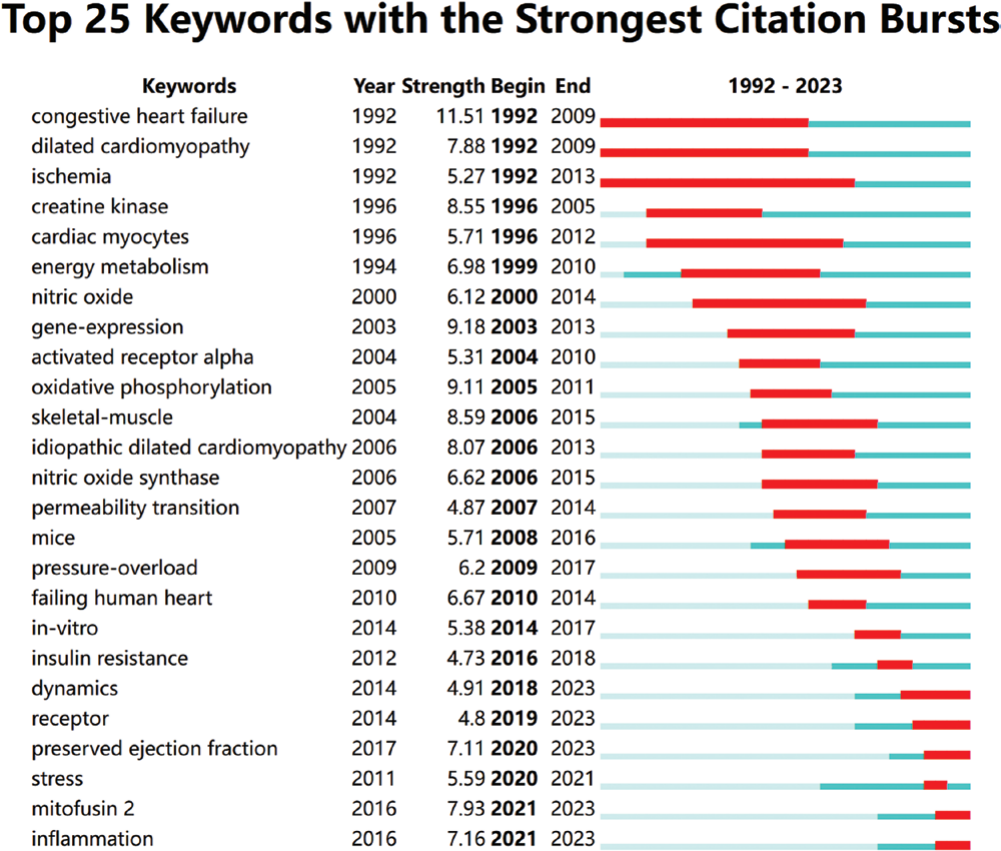
Keyword burst.

Keyword burst analysis can reflect the research frontiers in a certain period and reveal the evolving trends of exploration in the field. By using the Burstness function in CiteSpace, burst keywords were detected (Fig. 7). Based on the chronological order and combined with the annual publication volume, the research themes in this field were divided into 3 stages, including Initial Stage (1993–2003), in which the research themes focused on specific diseases such as congestive heart failure, dilated cardiomyopathy, ischemic lesions, and elevated creatine kinase; Steady Development Stage (2004–2011), in which the research themes focused on pathological mechanisms (including oxidative phosphorylation, insulin resistance, and mitochondrial permeability transition), and relevant targets and gene expression levels including activated receptor alpha and nitric oxide synthase, and such research encompassed in vivo animal studies and in vitro cell studies; and Rapid Development Stage (2012–2023), in which the research themes mainly revolved around oxidative stress, inflammatory responses, relevant proteins, and potential approaches for disease prevention and treatment. The research topics such as “preserved ejection fraction,” “stress,” “mitofusin2,” and “inflammation” remained popular. It is predicted that heart failure with preserved ejection fraction (HFpEF) will be a key focus in the future, and the study on the key mitochondrial dynamics proteins and inflammation-related signaling pathways will be the main research directions in the field of mitochondrial dynamics in HF.

### 3.4. Citation analysis of literature/journals

#### 3.4.1. Citation analysis of literature.

The analysis of cited literature can demonstrate the knowledge foundation and internal structure of a research field. The top 10 cited articles are displayed in Table 7. Most of these articles were published between 2016 and 2019. The most frequently cited article was by Brown et al from Virginia Tech in the journal “NAT REV CARDIOL,”^[8]^ which was cited 62 times. This article was a consensus statement on mitochondrial function as a therapeutic target for HF, which provided insights into the mechanisms of mitochondrial dysfunction in HF, including impaired mitochondrial electron transport chain activity, increased ROS production, altered substrate utilization, abnormal mitochondrial dynamics, and changed ion homeostasis. The article also outlined the emerging therapeutic strategies targeting mitochondria to improve cardiac function in HF, laying a certain foundation for the research in this field. The second most frequently cited article was by Zhou and Tian from the Mitochondria and Metabolism Center at the University of Washington published in the journal “J CLIN INVEST.”^[18]^ This article was a comprehensive review of mitochondrial dysfunction in the pathophysiology of HF, which systematically summarized the research findings of previous researchers and provided a theoretical reference for further exploration in this field.

**Table 7:** Top 10 cited literature on mitochondrial dynamics in heart failure related studies.

Rank	Title	Author	Year	Journal	Citations
1	Expert consensus document: Mitochondrial function as a therapeutic target in heart failure	Brown DA	2017	NAT REV CARDIOL	62
2	Mitochondrial dysfunction in pathophysiology of heart failure	Zhou B	2018	J CLIN INVEST	40
3	2016 ESC Guidelines for the diagnosis and treatment of acute and chronic heart failure: The task force for the diagnosis and treatment of acute and chronic heart failure of the European Society of Cardiology (ESC) Developed with the special contribution of the Heart Failure Association (HFA) of the ESC	Ponikowski P	2016	EUR HEART J	25
4	Metabolic remodeling in heart failure	Bertero E	2018	NAT REV CARDIOL	24
5	The failing heart relies on ketone bodies as a fuel	Aubert G	2016	CIRCULATION	22
6	Dapagliflozin in patients with heart failure and reduced ejection fraction	McMurray JJV	2019	NEW ENGL J MED	20
7	The failing heart—an engine out of fuel	Neubauer S	2007	NEW ENGL J MED	19
8	AMPKα2 protects against the development of heart failure by enhancing mitophagy via PINK1 phosphorylation	Wang B	2018	CIRC RES	19
9	Diabetic cardiomyopathy: An update of mechanisms contributing to this clinical entity	Jia GH	2018	CIRC RES	18
10	Unlocking the secrets of mitochondria in the cardiovascular system: Path to a cure in heart failure—a report from the 2018 National Heart, Lung, and Blood Institute workshop	Tian R	2019	CIRCULATION	17

#### 3.4.2. Co-citation analysis of journals.

Table 8 presents the top 10 journals ranked by the co-citation frequency, along with their Impact Factors (IF, IF2021, the latest official data published in 2022), and Journal Citation Reports (JCR) quartiles for 2021. Both the IF and JCR quartiles reflect the influence and status of the journals. Among the top 10 journals in terms of their co-citation frequency, 5 were in the Q1 quartile of JCR, and 4 were in the Q2 quartile. “CIRCULATION” had the highest citation frequency. These findings indicate that the following journals have a significant impact on the field of mitochondrial dynamics in HF. To learn and stay updated on the research progress and cutting-edge information in this field, it is recommended to refer to the publications in these aforementioned journals.

**Table 8:** Top 10 journals with total citations of articles and their JCR partitions and impact factors.

Rank	Journal	Citations	JCR (2023)	IF (2023)
1	CIRCULATION	1039	Q2	7.4
2	CIRC RES	966	Q1	20.1
3	J MOL CELL CARDIOL	824	Q2	5.0
4	CARDIOVASC RES	815	Q1	10.8
5	J BIOL CHEM	794	Q2	5.48
6	AM J PHYSIOL-HEART C	780	Q2	5.1
7	P NATL ACAD SCI USA	728	Q1	11.1
8	J AM COLL CARDIOL	689	Q1	24
9	J CLIN INVEST	673	Q3	5.5
10	NATURE	551	Q1	64.8

**Figure 8.**
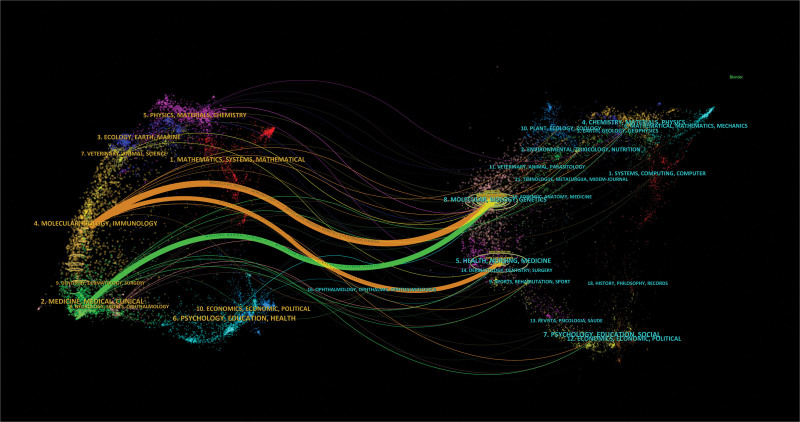
Clustering relationship of journal analysis.

By applying the method of knowledge flow analysis, the evolutionary process of citation and co-citation among journals was explored in this study.^[17]^ The overlapping dual-graph of journals reveals the distribution of academic topics, changes in citation trajectories, and shifts in research centers, as shown in Figure 8.^[19]^ The left labels in the dual mapping represent the citing domains, while the right labels stand for the cited domains. The connections were merged using the Z-score function. Colored curved lines originating from the citation graph and pointing towards the cited graph represent the contextual citation paths. As shown in the figure, there were primarily 3 citation paths. The green citation lines suggested that research from medical and clinical journals was frequently cited by molecular, biological, and genetic journals. While the 2 orange citation lines demonstrated that research from molecular, biological, and immunological journals was frequently cited by molecular, biological, genetic, nursing, and medical journals.

## 4. Discussion

HF, a complex clinical syndrome, results from structural or functional abnormalities of the heart due to various reasons. Multiple studies have shown that the 5-year survival rate of HF patients is only around 50%, and the incidence of HF keeps increasing year by year. It is estimated that the population of HF patients in China will reach 20 million by 2030.^[20]^ Currently, the recognized mechanisms related to the pathogenesis of HF mainly include excessive activation of the neurohumoral system, immune dysregulation, energy metabolic disorders, and oxidative stress damage. Of these mechanisms, both energy metabolism and oxidative stress damage revolve around mitochondria, highlighting the central role of mitochondria in HF.

The balance of mitochondrial dynamics is the prerequisite for maintaining the mitochondrial structural integrity, and the dynamic balance between mitochondrial fission and fusion is the basis for keeping mitochondrial homeostasis.^[6]^ According to our results in this study, mitochondrial dynamics interventions in HF research mainly focus on congestive heart failure, ischemic heart disease, dilated cardiomyopathy, and HFpEF. The pathological processes involved include oxidative stress, inflammatory responses, insulin resistance, and mitochondrial membrane permeability. Besides, the related factors include inducible nitric oxide synthase (iNOS), creatine kinase MB (CKMB), and mitochondrial fusion protein 2 (MFN2).

The findings in this study highlight the importance of mitochondrial dynamics in HF research, particularly in the context of energy metabolism and oxidative stress-related mechanisms. Therefore, understanding and targeting mitochondrial dynamics may provide potential therapeutic strategies for HF. Furthermore, more investigations in this field are warranted to elucidate the precise mechanisms and explore novel interventions for the better management of HF.

### 4.1. Research publication landscape of mitochondrial dynamics in HF

By conducting CiteSpace analysis on the literature related to mitochondrial dynamics in HF, an overall increasing trend in the publication volume was observed, indicating that growing attention was paid to mitochondrial dynamics research in HF. This field is in a vigorous development period, and the research interest is expected to continue to rise. The United States ranked first in terms of research publications in the field of mitochondrial dynamics in HF. Meanwhile, the complex co-occurrence network diagram suggested that preliminary collaborative relationships were established among institutions from various countries. However, close collaborations between different countries are currently limited to a few economically and technologically advanced nations, suggesting a relatively weak intensity and depth of research collaboration between countries. It is recommended to establish specialized academic exchange programs between countries to promote communication and collaboration among international institutions. By leveraging complementary strengths, more fruitful research outcomes can be achieved, and more practical and effective methods for the prevention and treatment of HF can be provided.

The majority of research publications in this field were concentrated in medical universities and some comprehensive universities. The University of California, with the highest publication volume, had close connections with other research institutions. However, overall, there was still a lack of a global collaborative network among different institutions, hindering the long-term stable development of this field. Therefore, it is strongly recommended that research institutions from different countries conduct more comprehensive and in-depth collaborative research to promote a global research network for mitochondrial dynamics in HF. As for authors, the influential figures in this field included E. Dale Abel, Heiko Bugger, and others. Paying attention to the achievements of these authors will help gain a better understanding of the cutting-edge dynamics and development trends in this field.

Most of the research concerning mitochondrial dynamics was published in the *Journal of Molecular and Cellular Cardiology* (n = 79 [4.5%]). With an IF of 5.475 (Q2), it is the most common journal in this field but not the journal with the highest IF. Among the co-cited journals, Circulation had the highest number of citations and was the most influential journal. The majority of co-cited journals were Q1 and Q2 journals, providing high-quality literature support for mitochondrial dynamics-related research. Furthermore, as shown by the overlapping dual-journal mapping (Fig. 8), “medical, clinical, molecular” was frequently cited in “molecular, biology, genetics,” indicating that current research on mitochondrial dynamics in the HF field increasingly focused on basic research.

### 4.2. Evolution of research focus in mitochondrial dynamics in HF

Studying the research focus and frontier keywords helps understand the focal points and evolution of a specific research field. Based on the analysis of keyword co-occurrence networks and clustering, along with the information provided in Tables 5 and 6, the main aspects of the evolving research focus in mitochondrial dynamics in HF were summarized as follows, Regulation Mechanisms of Mitochondrial Dynamics in HF with Different Etiologies, Functional Impairment and Energy Metabolism in Mitochondrial Dynamics in HF, Mitochondrial Fission and Fusion Proteins, and Inflammatory Responses and Oxidative Stress.

#### 4.2.1. Regulatory mechanisms of mitochondrial dynamics in HF differed based on different etiologies.

In the pathogenesis of diabetic cardiomyopathy-induced HF, the increased mitochondrial fission is mainly ascribed to the elevated blood glucose levels resulting from insulin resistance or insufficient insulin secretion, leading to disruption of the mitochondrial network structure. The mitochondrial dynamics-related proteins Drp1, Mfn2, and OPA1 are all regulated by the insulin signaling pathway. While dysregulation of these proteins by the insulin signaling pathway causes mitochondrial fragmentation and cellular dysfunction and failure.^[21]^ Under high glucose stimulation, the activation of ERK1/2 and ROCK1 induces phosphorylation of Drp1, down-regulation of OPA1, and loss of Mfn2, leading to mitochondrial fission and ROS generation, eventually triggering mitochondrial dysfunction and myocardial insulin resistance.^[22]^ The role of mitochondrial dynamics in regulating the insulin resistance mechanisms requires further exploration. In ischemic heart disease-induced HF, anaerobic glycolysis can result in the opening of the mitochondrial permeability transition pore (MPTP), reduction of ATP synthesis, overload of mitochondrial calcium, and loss of mitochondrial membrane potential. This further promotes the release of cytochrome C and apoptotic factors, thereby mediating changes in mitochondrial dynamics and inducing myocardial cell apoptosis.^[23]^ Consequently, manipulating mitochondrial dynamics plays a crucial role in controlling mitochondria in different types of HF and can provide strategies for optimizing the design of treatments for heart diseases.

#### 4.2.2. Application of mitochondrial dynamics in functional impairment and energy metabolism in HF.

Mitochondria exert a crucial effect on maintaining cardiac homeostasis, which is achieved by providing the main energy required for cardiac excitation-contraction coupling and controlling key intracellular survival and death pathways. Furthermore, they are involved in various aspects, including mitochondrial oxidative stress, mitochondrial biogenesis, mitochondrial dynamics, mitochondrial autophagy, and intracellular calcium regulation.^[24]^ Relevant studies have reported that the levels of ATP, phosphocreatine (PCr), and the PCr/ATP ratio are significantly reduced in cells from both human and animal models of HF, demonstrating the altered energy metabolism in failing hearts.^[25]^ Abnormal mitochondrial dynamics can lead to mitochondrial dysfunction, which then disrupts energy metabolism, ultimately causing irreversible deterioration of cardiac function.^[26]^ Based on these findings, widespread attention has been paid to the targeted regulation of mitochondrial dynamics to improve cardiac function and energy metabolism, which may become an effective strategy for the prevention and treatment of HF.

#### 4.2.3. Mitochondrial fission and fusion proteins.

As discovered in some studies, lipid overload creates an intracellular environment that promotes the acetylation of Drp1, which then enhances its activity and mitochondrial translocation, leading to cardiac cell dysfunction and necrosis. Therefore, Drp1 may be a key mediator of lipid overload-induced cardiac dysfunction and a potential therapeutic target for HF.^[27]^ With regard to mitochondrial fusion, the deficiency of Mfn1 and Mfn2 in the adult heart can inhibit the MPTP opening and reduce the infarct size, thus exerting a protective effect on the heart.^[28]^ In addition, the promoted fatty acid metabolism in the myocardium can up-regulate the i-AAA protease Yme1L, which improves cardiac function in the context of pressure overload-induced HF via regulating the Opa1-mediated mitochondrial dynamics balance.^[29]^ In mice with HF induced by ascending aortic constriction, treatment with the DRP1 inhibitor mdivi-1 can improve cardiac dysfunction by various mechanisms, such as regulating ventricular remodeling, inhibiting the expression of antiangiogenic factors, promoting angiogenesis, and suppressing mitochondrial fission.^[30]^ Therefore, targeted regulation of mitochondrial fusion and fission proteins has the potential to protect cardiac structure and function, finally improving the treatment of HF caused by ischemic heart disease.^[31]^

#### 4.2.4. Impact of inflammation and oxidative stress on mitochondrial dynamics in HF.

Excessive mitochondrial fission can lead to impaired mitochondrial respiratory function and exacerbate the accumulation of ROS in mitochondria. Consequently, excessive ROS can damage mitochondrial DNA (mtDNA), impair mitochondrial proteases, and induce lipid peroxidation, thereby triggering oxidative stress. On the other hand, inflammatory mediators, including ROS, nitric oxide (NO), interleukins (ILs), and tumor necrosis factor-alpha (TNF-α), can induce mitochondrial fusion and fission.^[32]^ HF can elevate the nitric oxide synthase (NOS) level, which in turn induces the production of excessive NO. NO, as a physiological vasodilator, can inhibit oxidative phosphorylation by binding to subunits of the respiratory complex I, thus reducing ATP synthesis. Besides, NO can also activate Drp1, which then promotes mitochondrial dynamics imbalance, causes an imbalance between ROS generation and elimination, places cells in an oxidative stress state and accelerates cell apoptosis.^[33]^

### 4.3. Trends in the study of mitochondrial dynamics in HF

#### 4.3.1. The involvement of mitochondrial dynamics in the HFpEF pathogenesis.

Compared with heart failure with reduced ejection fraction, treatment strategies for HFpEF have not reliably displayed improvements in hard outcomes. Current guidelines have recommended symptom improvement (using diuretics) and control of underlying conditions, especially for hypertension and atrial fibrillation.^[34]^ Despite extensive clinical research, effective treatment options for HFpEF are lacking. In key trials such as I-PRESERVE^[35]^ and CHARM-preserve,^[36]^ inhibition of the renin-angiotensin system failed to demonstrate improvement in the mortality rate of HFpEF patients. Chaanine et al^[37]^ found increased levels of the mitochondrial fission protein DRP1 in heart failure with reduced ejection fraction patients relative to both normal and HFpEF patients, while levels of OPA1 and MFN2 were not significantly different. Bode et al^[38]^ studied a metabolic HFpEF rat model and observed increased mitochondrial fission, consistent with the results obtained from multi-omic analyses in a rat model of HFpEF induced by hypertension. Studies have suggested that inflammation and exacerbated mitochondrial fission are 2 major biological processes involved in myocardial cell failure.^[39]^ Therefore, it is expected that future investigations will explore the pathogenesis of HFpEF from the perspective of mitochondrial dynamics and identify novel targets for disease prevention and treatment, thus clarifying the molecular mechanisms of targeted therapies.

#### 4.3.2. The relationship between mitochondrial dynamics, oxidative stress, and inflammation.

Mitochondrial oxidative stress, through damaging mitochondrial dynamics, increases the phosphorylation of DRP1, reduces the expression of MFN2, and promotes the synthesis of pro-inflammatory and pro-fibrotic cytokines, the activation of fibroblasts, and the accumulation of extracellular matrix, ultimately leading to interstitial fibrosis and passive stiffening of the myocardium, and resulting in diastolic dysfunction. Diastolic dysfunction is precisely the pathogenesis of HFpEF.^[40]^ The relationship between inflammation, oxidative stress, and mitochondrial dynamics is complex, and the underlying mechanisms remain to be further explored. Therefore, it is speculated that targeting the expression levels of inflammatory mediators in cells and enhancing the level of mitochondrial dynamics to increase the cellular antioxidant capacity and improve cardiac function will be important directions for future targeted therapies.

## 5. Conclusions

In summary, based on a literature analysis, this study summarizes the current research status, evolution, and latest development in mitochondrial dynamics in HF up to October 2023, and analyzes the prospects and trends in this field. Currently, research on mitochondrial dynamics in HF is under vigorous development. It is a developing trend in this research filed to explore differential gene expression in mitochondrial dynamics in HF and the molecular mechanisms of targeted therapies. Besides, investigating the pathogenesis of HFpEF through mitochondrial dynamics may be a future direction for research and warrants further exploration and investigations.

## Author contributions

**Conceptualization:** Sihan Jia, Juju Shang.

**Formal analysis:** Sihan Jia.

**Funding acquisition:** Sinai Li, Juju Shang.

**Software:** Sihan Jia, Yanjie Lian.

**Supervision:** Hongxu Liu, Juju Shang.

**Visualization:** Sihan Jia, Yanjie Lian.

**Writing – original draft:** Sihan Jia, Yanjie Lian.

**Writing – review & editing:** Sinai Li, Hongxu Liu, Juju Shang.
